# Rosiridin Attenuates Scopolamine-Induced Cognitive Impairments in Rats via Inhibition of Oxidative and Nitrative Stress Leaded Caspase-3/9 and TNF-α Signaling Pathways

**DOI:** 10.3390/molecules27185888

**Published:** 2022-09-10

**Authors:** Muhammad Afzal, Sami I. Alzarea, Khalid Saad Alharbi, Abdulaziz I. Alzarea, Sattam Khulaif Alenezi, Mohammed Salem Alshammari, Ali H. Alquraini, Imran Kazmi

**Affiliations:** 1 Department of Pharmacology, College of Pharmacy, Jouf University, Sakaka 72341, Aljouf, Saudi Arabia; 2 Department of Clinical Pharmacy, College of Pharmacy, Jouf University, Sakaka 72341, Aljouf, Saudi Arabia; 3 Department of Pharmacology and Toxicology, Unaizah College of Pharmacy, Qassim University, Buraydah 52571, Qassim, Saudi Arabia; 4 Department of Pharmacy Practice, Unaizah College of Pharmacy, Qassim University, Buraydah 52571, Qassim, Saudi Arabia; 5 Department of Pharmaceutical Chemistry, Faculty of Clinical Pharmacy, Al Baha University, Al Baha 65779, Al Baha, Saudi Arabia; 6 Department of Biochemistry, Faculty of Science, King Abdulaziz University, Jeddah 21589, Makkah, Saudi Arabia

**Keywords:** acetylcholinesterase, choline acetyltransferase, neuroprotective, rosiridin

## Abstract

Aim: A monoterpene and bioactive component of the plant *Rhodiola rosea* (*R. rosea*), rosiridin has beneficial effects on the human central nervous system and enhances brain function. The goal of this scientific study was to determine if rosiridin might shield rats from neurocognitive problems induced by scopolamine. Methods: To track the potential toxicities in rats, the acute toxicity in rats was clarified. Rosiridin at a dose of 10 mg/kg was tested in rats for 14 days. At the conclusion of the investigation, behavioral parameters that were used to identify the rats’ cognitive and motor abilities were evaluated. Several biochemical parameters were estimated using the prepared homogenate, including acetylcholine esterase (AChE), choline acetyltransferase (ChAT), radical scavengers produced by the body (Catalase-CAT, superoxide dismutase-SOD, and reduced glutathione-GSH), indicators of oxidative and nitrative burnout, pro-inflammatory (Interleukins- IL-1β, IL-6, interferon gamma IFN-ꝩ, and tumor necrosis factor-TNF-α), and cell apoptosis caspases 3 and 9. Results and Conclusion: A significant behavioral parameter restoration was seen in the rosiridin-treated group, including reduction in latency time during acquisition and retention trial in the Morris water maze test, and percentage of spontaneous alterations in the y-maze test, when compared to the disease control group that received scopolamine; rosiridin also altered the oxidative stress and neuroinflammatory markers, as well as restoring Ach and ChAT activities and normalizing GSH, SOD, MDA, TNF-α, nitrate, IL-1β, IL-6, IFN-ꝩ, caspases 3 and 9 levels. The results imply that rosiridin limits the effect of scopolamine on rat cognitive function.

## 1. Introduction

Cognitive abilities are built on the foundations of memory and learning [1]. Neurodegenerative diseases, which are becoming more frequent as individuals become older, are associated with cognitive impairment [2]. It is now a significant worldwide public health concern [3]. Memory impairment in Alzheimer’s disease (AD) is marked owing to a surge in β amyloid, alterations in the cholinergic network, phosphorylated tau protein, and permanent cognitive impairment [2]. The number of citizens dealing with cognitive impairments has steadily increased with the passage of time, but the condition remains so crippling that it tends to become a huge social and financial liability on society [4]. The therapies for the ailment include the drugs that block or inhibit cholinesterase and N-methyl-D-aspartate binding site antagonists that are used to reduce or delay AD symptoms [5,6]. However, there is currently no cure for the condition. As a result, scholars everywhere across the world are looking for novel ways to address AD in a safe and efficient manner [6]. As a consequence, there is now extensive medicinal development concerning the memory-enhancing qualities of herbal remedies [7].

The muscarinic cholinergic receptor antagonist, scopolamine, promotes memory impairment and partially imitates AD and dementia [8]. Allied memory deficit is among the most often utilized pharmacological models in learning and memory research [9]. It inhibits the propagation of neural signals by blocking muscarinic acetylcholine receptors, causing neurocognitive problems [1,9]. Several cognitive animal models have been developed to examine cognitive characteristic, each having its unique path and physiological underpinning [10]. Scopolamine inhibits memory and learning in rats by altering central cholinergic processes. It operates as a competitive antagonist to block muscarinic receptors [10,11]. Scopolamine, as an anticholinergic, prevents ACh from attaching to receptors, resulting in an increase in Ach [12]. As a result, the hippocampal nerves are damaged, leading to cognitive decline and learning difficulties [13]. This is analogous to how AD causes the death of cortical cholinergic neurons and reduced central cholinergic function [13].

In laboratory development, herbal remedies and synthetic molecules with anti-inflammatory and antioxidant properties, and molecules that alter the cholinergic network have been found to be neuroprotective [14,15,16]. The largest family of plant secondary metabolites, monoterpenes, which are composed of hydrocarbons, are frequently found in essential oils. The discovery and manufacturing of novel physiologically active chemicals largely depends on monoterpenes and their derivatives [17]. It has been discovered that monoterpenes have antidiabetic [18,19], cardioprotective [17,20,21], anti-inflammatory [17,22], antioxidant [23], antihyperlipidemic [24], anticancer [17,25], antimicrobial [17,26], antiviral [17,25], immunomodulatory [17,25], and antispasmodic actions [17,25]. Monoterpenes have lately attracted much attention for their ability to prevent age-related neurodegeneration and modulate neuronal activity [27]. Human and animal dietary supplementation with monoterpene-rich plant or food extracts has been shown to improve cognitive function, implying that susceptible neurons are protected, existing neuronal function is enhanced, and neuronal regeneration is stimulated [28]. Monoterpenes decrease inflammatory mediators, boost antioxidant enzymes, reduce oxidative damage, and modify gene expression levels in neurological disorders [29]. In several models of neurodegenerative disorders, several monoterpenes have been shown to exhibit neuroprotective therapeutic potential [29,30]. Rosiridin is a monoterpene and bioactive element of the plant *Rhodiola rosea* (R. rosea) with remarkable monoamine inhibitory potential and is beneficial in the management of depressive episodes and early onset dementia [31,32,33]. The root of Rhodiola species (Crassulaceae) is used in traditional remedies in the northeast Asian region as an antiasthmatic, a bleeding cure, and an antiaging therapy [34,35]. Previous research has demonstrated that Rhodiola root water extracts have beneficial effects on the human central nervous system and enhance brain function [36,37]. In addition to being antioxidants, they are also considered to be good for one’s health [38]. According to previously published research, monoamine oxidase (MAO) activation has a crucial role in the pathogenesis of AD, including the creation of amyloid plaques from Aβ production. Rosiridin can be used to treat early-onset dementia and despair because it inhibits monoamine oxidase A and B [39]. Rosiridin has recently been found to have an anti-Huntington’s effect through oxidative stress/acetylcholine esterase (AChE), and inhibition and modulation of succinate dehydrogenase, nitrite, and brain-derived neurotrophic factor levels against 3-nitropropionic acid in rodents [39]. However, no research has been completed on the impact of rosiridin on cognitive impairment caused by scopolamine. As a result, the main goal of this study was to determine if rosiridin may help with scopolamine-induced amnesia.

## 2. Scientific Methods

### 2.1. Chemicals

Scopolamine and rosiridin were used in this study (Sigma-Aldrich, St. Louis, MI, USA). High-quality reagents and chemicals were used in the experiment.

### 2.2. Animals

Male adult Wistar rats (*n* = 6) weighing 200 ± 20 g (aged 8 weeks) were obtained and maintained under the laboratory settings specified by the CPCSEA recommendations, which comprise a humidity range of 40–50%, a temperature range of 23 ± 2 °C, and a 12–12 light–dark cycle. The rats were housed in polypropylene cages, and they had access to unlimited amounts of food pellets and water.

### 2.3. Acute Oral Toxicity

The Organization for Economic Cooperation and Development (OECD) recommendations for acute oral toxicity (LD_50_) of rosiridin were followed (ANNEX423). For the acute oral toxicity, rosiridin was orally administered to the rats at the maximum dosage. However, no abnormalities were discovered. We chose a treatment dosage of 10 mg/kg based on the results of this trial.

### 2.4. Experimental Design

To elicit cognitive deficits in animals, 1 mg/kg i.p. scopolamine was injected [40,41]. Rosiridin was administered orally to animal for fourteen days.

**Figure 1:**
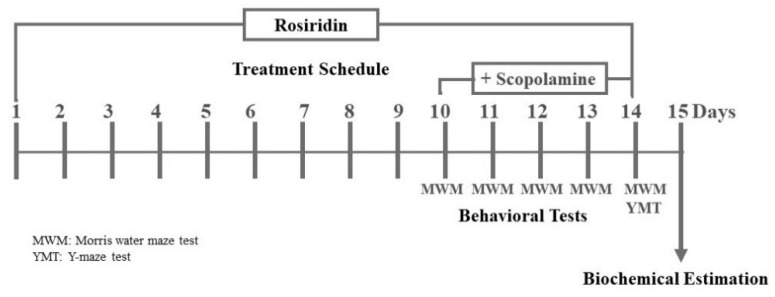
Experimental design.

A combination of 24 animals were allocated to each of 4 categories and given the following condition: Group 1 (saline control) and Group 2 (scopolamine control) were orally given 0.5% sodium carboxy methyl cellulose vehicle 3 mL; Group 3 were given rosiridin 10 mg/kg (rosiridin oral treatment); and Group 4 were given rosiridin 10 mg/kg per se, respectively (rosiridin per se). Day 10–14 (5 days) of the 14-day treatment plan, 1 h after the abovementioned oral treatments, Group 1 was given i.p. standard saline solution 3 mL/kg/day and scopolamine 1 mg/kg (i.p.) was administered to Group 2 and 4. Behavioral assessments for rats were completed 2 h after scopolamine therapy during the medication regime. Animals were slaughtered and brains were retrieved for biomarkers on day 15 following behavioral investigations (Figure 1).

### 2.5. Behavioral Parameters to Screen for Memory Impairments

#### 2.5.1. MWM (Morris Water Maze) Examination

The MWM examination was measured using the method proposed by Aksoz et al., 2019 [42]. The MWM round tank was split into four exactly equivalent quadrants or zones. For the initial four days, an escape platform was kept 1 cm beneath the liquid of any one of the quadrants. Small white materials were strewn across the surface of the liquid. One of the animals was placed in one of randomly sampled spots in the tank on each day of the learning exercise (three tests per event). The animal was placed in the tank to begin the experiment. Whenever the animal discovered and stepped onto the platform, the experiment was called off, and the average escape latency was calculated. The highest exposure time was 60 s. If the animal did not reach onto the platform within 60 s, it was initially pushed upon this platform and 60 s escape latency was noted. The animal was retained on the stand for 20 s between sessions. The animals were delicately wiped and put in respective home cages just after test was conducted at all three beginning points. The rat’s memory of the position of the concealed platform for 60 s was assessed during 5th day of ”probe trial”. During this phase, the platform was removed from the tank. Thus, the latency time to find the proper quadrant where the platform was previously and the time spent in this compartment were recorded.

#### 2.5.2. Y-Maze Test

The Y-maze test was carried out according to the instructions in Djeuzong et al., 2019 [40]. The Y-maze test, which recorded random rearrangements, was used to examine the animals’ working memory. A wooden maze with three independent arms (40 cm long, 35 cm tall, and 12 cm wide) was used, each staggered by a 120° inclination. To distinguish them, the walls of each arm were adorned with various designs and named X, Y, and Z. Rats were individually placed at the end of a maze’s branch for free exploration. The sum of visits at each arm was counted throughout the course of 5 min. To mitigate smells, the device was cleaned with 10% ethanol after every exercise. Three sequential entries in three separate arms, such as XYZ, ZXY, or YZX, were defined as a random alternation.

### 2.6. Parameters of Biochemistry

#### 2.6.1. Excising a Brain Tissue

Animals were sacrificed following ketamine (80 mg/kg) and xyline (16 mg/kg) anaesthesia, and the entire brain was taken and preserved at a temperature of below −50 °C [42].

#### 2.6.2. Creating a Homogenate from Brain Cells

The animals’ brains were thoroughly rinsed with ice-cold physiological saline. Phosphate buffer of neutral pH was used to consolidate cerebral samples. The samples were centrifuged, and the supernatant was employed for biochemical testing.

### 2.7. Neurochemical Quantification

#### 2.7.1. Cholinesterase (AChE) and Transferase (ChAT) Functioning

To assess AChE activity expressed as µM/mg protein, a technique identical to that published by Ellman et al., (1961) was used [43,44]. ChAT levels in brain tissue were tested using commercial kits.

#### 2.7.2. Biological Scavengers

Ellman measured reduced glutathione (GSH) using a method that had previously been described [45]. The Misra and Frodvich approach were used to assay the superoxide dismutase (SOD) [46,47,48]. The Catalase activity was measured according to the technique reported by Afzal et al., 2021 [15,46].

#### 2.7.3. Sensors of Oxidative and Nitrative Stress

The Wills technique was used to calculate malondialdehyde (MDA) in brain homogenate. The MDA concentration was expressed as nM/mg protein [47]. The calorimetric approach was used to determine nitrite, a measure of nitrite generation [48]. A sodium nitrite calibration graph was used to determine the nitrite content, and the results were expressed in nanograms per milligram of protein [45,49].

#### 2.7.4. Cytokines That Promote and Inhibit Inflammation

An immunoassay kit was used to measure the proinflammatory cytokines tumor necrosis factor (TNF-α), interleukins(IL-6), IL-1β, and interferon gamma (IFN-ꝩ) expression. Calibration curves were used to calculate indicator concentrations, which were then stated in pg/mL protein.

#### 2.7.5. Programmed Cell Death Indicators

The caspases 3 and 9 were substantiated using an ELISA kit. The levels of these suicide biomarkers were assessed in nanograms per milliliter.

### 2.8. Statistical Analytics

Graph Pad Prism was used to examine the data. The information is presented in the form of median standard deviation of the mean (S.E.M.). Morris water maze assessment were performed using two-way analysis of variance (ANOVA) followed by the Bonferroni post analytic test, and other assessment was done by one-way ANOVA by Tukey’s analytic test. The relevance threshold was established at *p* < 0.05.

## 3. Results

### 3.1. MWM Acquisition Process

**Figure 2:**
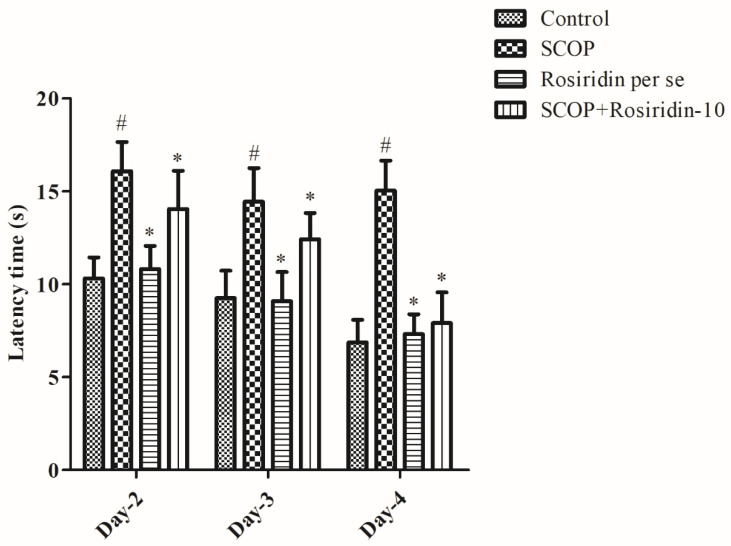
Depicts the effect of the rosiridin during the Morris water maze acquisition process. Mean ± S.E.M. (*n* = 6). # *p* < 0.001 vs. normal control, * *p* < 0.05 vs. scopolamine control. Two-way ANOVA followed by Bonferroni post analytic test.

Animal cognition was hindered by scopolamine ingestion, as seen by situation escalating to disengage from floating and approach the static platform in MWM. Figure 1 depicts the effect of the rosiridin during the acquisition process. Scopolamine substantially (*p* < 0.001) provoked cognitive deficits in the untreated comparison animals on the fourth day of the acquisition process when compared with saline control. The rosiridin 10 mg/kg, on the other hand, demonstrated the greatest (*p* < 0.001) reduction in lag to approach the static platform (Figure 2).

### 3.2. MWM Retention Phase

**Figure 3:**
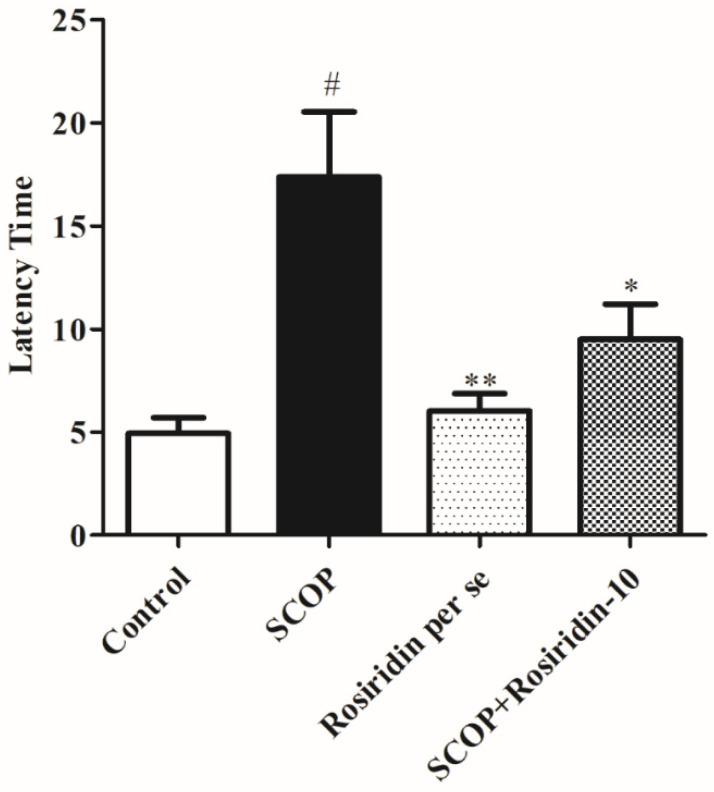
Graph depicts the effect of rosiridin during the Morris water maze retention period. Mean ± S.E.M. (*n* = 6). # *p* < 0.001 vs. normal control, ** *p* < 0.01 vs. scopolamine control, * *p* < 0.05 vs. scopolamine control; one-way ANOVA followed by Tukey’s test.

**Figure 4:**
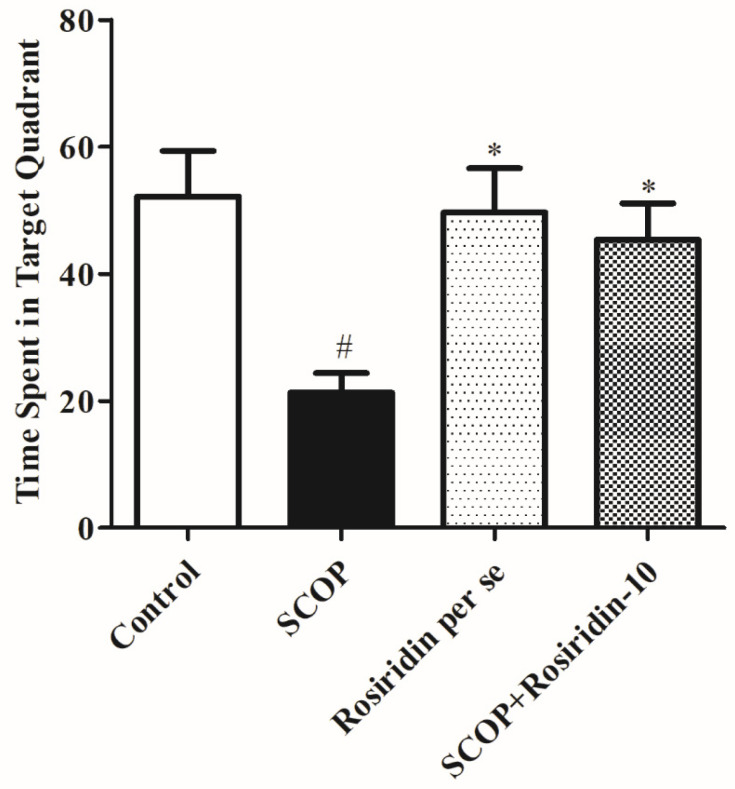
Graph depicts the time spent in the target quadrant. Mean ± S.E.M. (*n* = 6). # *p* < 0.001 vs. normal control, * *p* < 0.05 vs. scopolamine control; one-way ANOVA followed by Tukey’s test.

Figure 3 depicts the effect of rosiridin during the retention period. During the retention session, scopolamine treatment raised the latency to reach the platform substantially (*p* < 0.001) when correlated to the usual comparison; rosiridin significantly (*p* < 0.001) reduced the lag for approaching the station. In addition, correlated with saline control animals, the use of scopolamine as a negative control had a major impact (*p* < 0.001). During this phase, the time spent in the target quadrant was reduced (Figure 4). Rosiridin demonstrated the greatest (*p* < 0.001) increase in available time in the desired section.

### 3.3. The Y-Maze Examination of Spatial Working Memory

**Figure 5:**
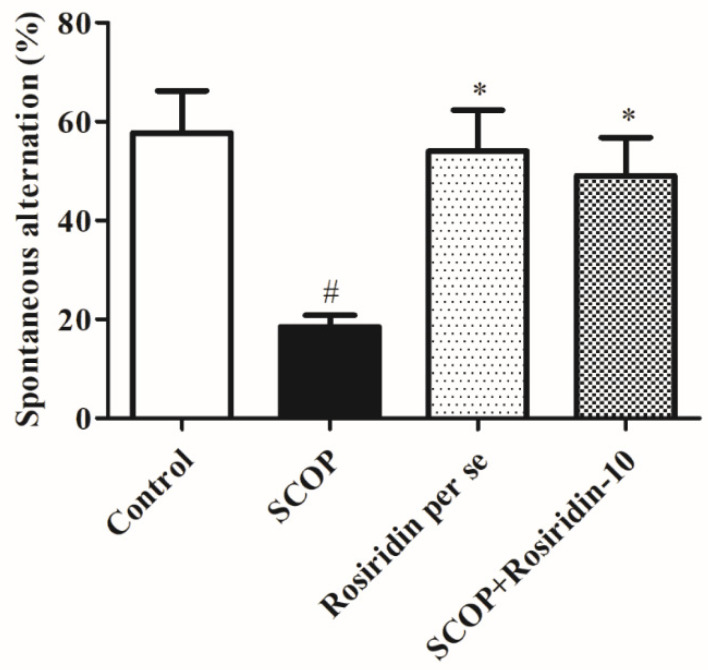
Graph depicts the effect of rosiridin in the Y-maze. Mean ± S.E.M. (*n* = 6). # *p* < 0.001 vs. normal control, * *p* < 0.05 vs. scopolamine control; one-way ANOVA followed by Tukey’s test.

Figure 5 represents the outcomes of the spatial working memory. Here, between negative controls and the classic control category, there was a substantial (*p* < 0.01) decrease in the number of random rearrangements. The rosiridin exhibited an insignificant (*p* > 0.05) rise in this metric.

### 3.4. Cholinesterase (AChE) and Transferase (ChAT) Functioning

**Figure 6:**
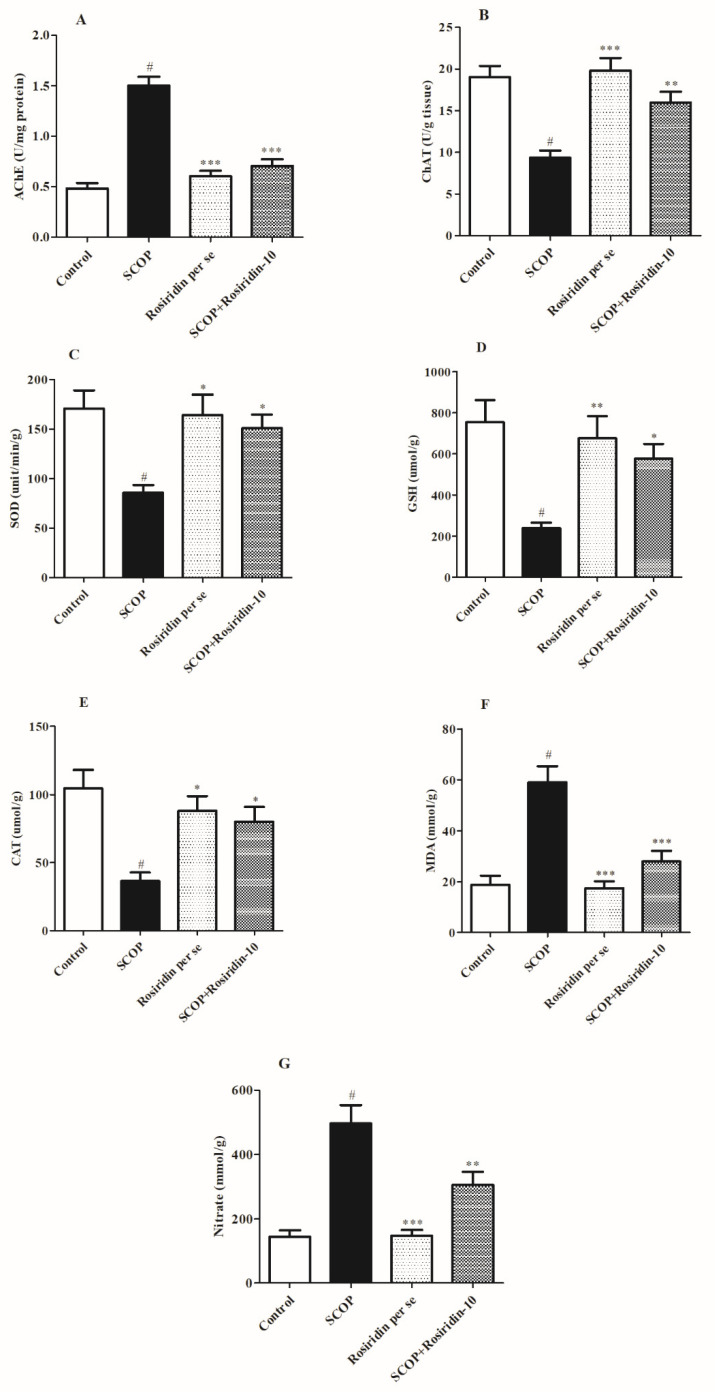
Graphs illustrating the effect of rosiridin on (**A**) AChE, (**B**) ChAT, (**C**) SOD, (**D**) GSH, (**E**) CAT, (**F**) MDA, and (**G**) nitrite level estimation. Mean ± S.E.M. (*n* = 6). # *p* < 0.001 vs. normal control, *** *p* < 0.001 vs. scopolamine control, ** *p* < 0.01 vs. scopolamine control, * *p* < 0.05 vs. scopolamine control; one-way ANOVA followed by Tukey’s test.

In correlation with the normal control, the scopolamine control had greater AChE (*p* < 0.05) and lower ChAT levels. When scopolamine-treated rats were given rosiridin, the levels of AChE (*p* < 0.001) and ChAT (*p* < 0.001) were considerably lower in correlation with control animals (Figure 6A,B).

### 3.5. Biological Scavengers

The levels of antioxidant defense (SOD, GSH, and CAT) were disrupted by scopolamine ingestion. Scopolamine control found significant reduction in biological scavengers such as SOD, GSH, and catalase than normal control rats (*p* < 0.05). Treatment of scopolamine-injected rats with rosiridin (10 mg/kg) restored GSH (*p* < 0.001), SOD (*p* < 0.001), and catalase (*p* < 0.001) levels to normal. Figure 6C–E illustrates the antioxidative defense level.

### 3.6. Stress-Level Assessment

In the brains of treatment animals, scopolamine elicited stress indicators MDA and nitrite. In scopolamine-treated rat, increased MDA and nitrite levels (*p* < 0.05) were detected. When contrasted with the scopolamine control group, rosiridin therapy reduced the elevated levels of MDA (*p* < 0.01) and nitrite (*p* < 0.001). Figure 6F,G shows the results of MDA and nitrite levels.

### 3.7. Neuromodulatory Cytokines

**Figure 7:**
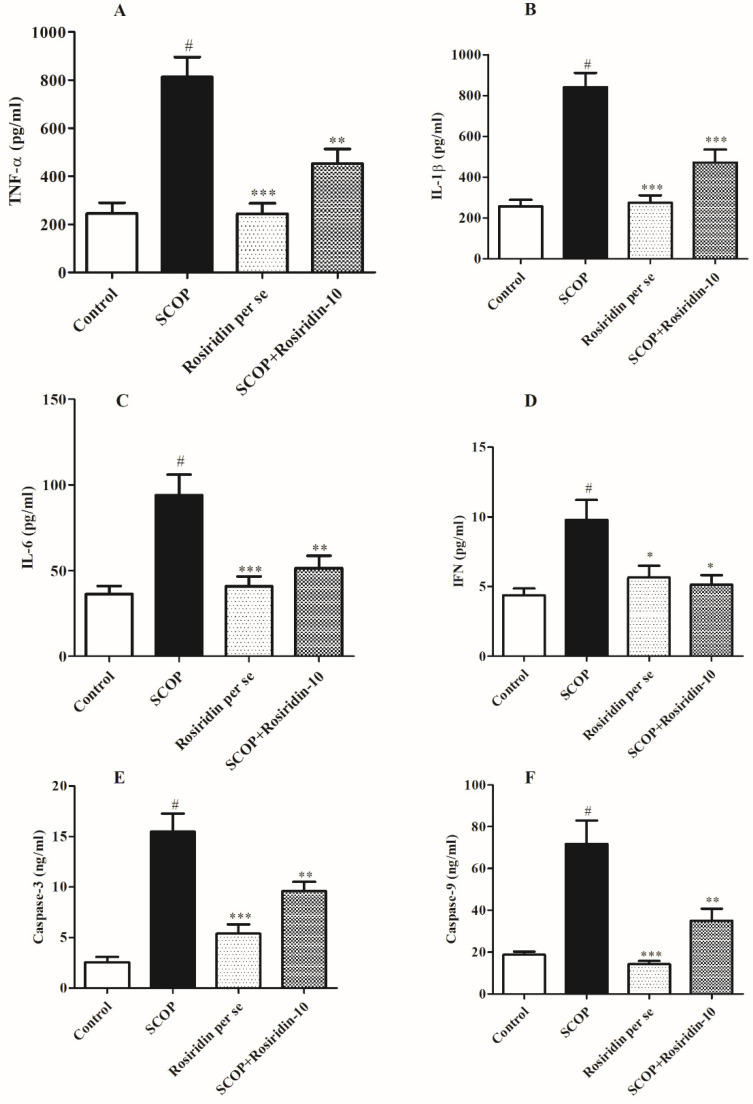
Graphs illustrating the influence of rosiridin on (**A**) TNF-α, (**B**) IL-1β, (**C**) IL-6, (**D**) IFN (**E**) caspase 3, and (**F**) caspase 9. Mean ± S.E.M. (*n* = 6). # *p* < 0.001 vs. normal control, *** *p* < 0.001 vs. scopolamine control, ** *p* < 0.01 vs. scopolamine control, * *p* < 0.05 vs. scopolamine control; one-way ANOVA followed by Tukey’s test.

When scopolamine control was correlated with saline control, proinflammatory cytokines TNF-α, IL-1 β, IL-6, and IFN-ꝩ were considerably raised (*p* < 0.01). When scopolamine control was given rosiridin (10 mg/kg), the levels of IL-1 (*p* < 0.01), IL-6 (*p* < 0.01), IFN (*p* < 0.01), and TNF-α (*p* < 0.01) were reduced. Figure 7A–D shows the findings of TNF-α, IL-1 β, IL-6, and IFN tests.

### 3.8. Programmed Cell Death Indicators

Figure 7E,F illustrates the influence of rosiridin on several apoptotic markers. When normal control correlated, the concentration of caspase 3 in the neuronal sample was considerably (*p* < 0.001) higher in the untreated. Furthermore, the concentration of caspase 9 was considerably restore (*p* < 0.001) in scopolamine control was given rosiridin (10 mg/kg).

## 4. Discussion

Based on the investigation, rosiridin, a monoterpene, appears to have the ability to prevent dementia associated with AD. In this observational study of rosiridin, the behavioral and biochemical function of scopalamine-induced memory impairment in rats was investigated systematically; our approach leads to several key observations.

The MWM is widely used to test animals for this specific memory impairment [40,42,50]. An effective and sensitive test for assessing hippocampal–spatial learning and reference memory is the MWM. It is also used to identify medications with antiamnesic characteristics, or medicines that stop, reverse, or lessen memory loss after a brain injury. The delay to locate the platform during the acquisition phase was reduced in the current investigation by the injection of scopolamine for 15 days (day 1 to day 4). Scopolamine, however, increased significantly on the fourth day of this phase compared to the usual group, suggesting that rats’ ability to learn may have been impaired. Scopolamine treatment increased the latency to find the platform and decreased the duration spent in the target quadrant on the fifth day of the experiment, twenty-four hours after the acquisition phase, indicating an impairment of the reference memory. The fact that scopolamine administration prolonged the escape latency suggests that the animals’ spatial memory and learning abilities were impaired. Rosiridin treatment of scopolamine administrated animals enhanced escape latency in the MWM test.

One of the earliest signs of AD is working memory problems, which can make AD patients forget the question they were just asked [51]. Cognitive recall was greatly decreased in scopolamine-treated rats. Another tool used to investigate memory rotation in animals is Y-maze [40,52]. The Y-maze paradigm, which is based on rats’ propensity to explore unfamiliar locations, is typically chosen to evaluate working memory deficits in rodents [53]. Therefore, typical animals choose to investigate a different arm of the maze than the one they previously visited. The proportion of spontaneous alternations in maze arm entrance dropped in rats given scopolamine and vehicle for 15 days, suggesting that the animal had forgotten the arm it recently visited. These findings showed that there may be a working memory deficit.

Memory retention was significantly diminished in scopolamine-treated rats. In comparison to scopolamine-untreated rats, rosiridin treatment improved memory retention in scopolamine-treated rats. The outcomes of the MWM and Y-maze tests indicate that rosiridin protects against allied cognitive decline. Using behavioral tests such as MWM and Y-maze, we first confirmed that rosiridin protects rats from scopolamine-induced spatial learning and memory deficits. These observations coincide with the findings of Djeuzong et al., 2021 [40].

Moreover, we reveal that rosiridin has protective capacity in the brain by considerably neutralizing the effects of scopolamine on MDA, GSH, Catalase, and SOD levels. Finally, we show for the first time that rosiridin lowered programmed cell death indicators caspases 3 and 9 levels that had been enhanced by scopolamine. Programmed cell death indicators caspases 3 and 9 are widely known for their role in pathophysiology of AD.

Memory deficits and behavioral abnormalities caused by scopolamine are indicated by spatial learning and memory. Scopalamine causes significant deterioration in cognitive function, which has been linked to increased AChE, oxidative stress, neuroinflammatory markers, IL-1β, TNF-α, IL-6, and IFN, in the brain. Treatment with rosiridin, on the other hand, restored the scopolamine-induced behavioral and metabolic changes. Rosiridin inhibited scopolamine impact on spatial cognitive performance and alterations in AChE. In the rat brain, rosiridin also restored endogenous antioxidant status, reducing neuroinflammatory indicators.

Learning and memory processes are connected to the central cholinergic system and to Ach, which is broadly distributed throughout the nervous system and a major neurotransmitter that influences intellectual performance and learning processes. ACh is digested in the synaptic space by AChE, which converts it to acetic acid and choline. Excessive AChE activity, on the other hand, can result in ACh deficiency and cognitive dysfunction. Simultaneously, ChAT can aid in acetylcholine biosynthesis [11]. As a result, cholinergic indicators such as AChE and ChAT expression are commonly used in the assessment of memory deficits [50]. In the brains of allied rats, rosiridin therapy drastically lowered AChE activity while increasing ChAT expression. According to the outcomes of the MWM and EPM examinations, rosiridin may maintain neurons via altering the integrity of cholinergic neuronal networks.

Oxidative and nitrative stresses are important factors in the aetiology and progression of neurodegenerative diseases [54,55,56]. When quantities of peroxides and reactive oxygen species (ROS) surpass natural antioxidant defenses, oxidative stress occurs [57]. Scopolamine, a physiological modulator of neurotransmitter acetylcholine, impairs learning and memory in laboratory rats [58]. Furthermore, because the brain has limited antioxidant defense systems, it is extremely vulnerable to oxidative damage [59,60,61]. In this study, rosiridin therapy decreased MDA and nitrate levels in the brain while increasing intrinsic defenders, including GSH, SOD, and catalase activity.

The cellular redox response system protects cells from oxidative stress by boosting the expression of neuroprotective enzymes that detoxify and mitigate the risk of cell damage caused by oxidative stress [62,63].

As a result, the transcription of proinflammatory cytokines is reduced [64,65]. The current study’s conclusions are strongly supported by the data. In scopolamine-treated rats, rosiridin reduced the levels of cytokines IL-1β, IL-6, and TNF-α.

In line with the findings of Demirci et al., scopolamine raised the levels of caspases 3 and 9, two important regulators of apoptosis, in brain tissue of the negative controls [66]. Scopolamine increases the synthesis of beta4-amyloidprotein (BAP) in the brain [67]. Via caspases 3 and 9, BAP causes neuronal cell death and pertains to AD genetic susceptibility [68]. Furthermore, this drug inhibits cell proliferation via boosting the synthesis of the apoptosis-inducing protein Bax, which triggers cell death by increasing the release of caspase activator in neuronal tissues. [69,70]. Scopolamine raised the levels of caspases 3 and 9 in the brain tissues in this investigation, corroborating the previous findings. The levels of caspase regulatory proteins determined by immunoassay in brain tissue were much lower after treatment with rosiridin, indicating a change in apoptotic processes. These findings also suggest that rosiridin has antiapoptotic action, which may contribute to its neuroprotective properties [49,71].

## 5. Conclusions

The current work shows that rosiridin, a monoterpene, reduces scopolamine-induced behavioral and biochemical aberrations in rats by reducing inflammatory response and caspases levels. Rosiridin antioxidant and anti-inflammatory properties might indicate a possible positive impact. However, further study is needed to determine if rosiridin can help those with neurodegenerative diseases.

## Data Availability

Not applicable.
